# Supplementary material for: Seroprevalence of Chlamydia trachomatis, herpes simplex 2, Epstein-Barr virus, hepatitis C and associated factors among a cohort of men ages 18–70 years from three countries
Source: PLoS One. 2021 Jun 22;16(6):e0253005. doi: 10.1371/journal.pone.0253005 (PMC8219163; doi:10.1371/journal.pone.0253005)
Supplement: S1 Table — (DOCX) [file pone.0253005.s001.docx]

**S1 Table.** A Comparison of the Baseline Characteristics for the Simple Random Sample and the Full Parent HIM Study Cohort

| **Characteristic** | **Full cohort**  **(n=4290)** | | **Sub-cohort**  **(n=600)** | |  |
| --- | --- | --- | --- | --- | --- |
|  | **n** | **%** | **n** | **%** | **p-value^a^** |
| **Country** |  |  |  |  |  |
| USA | 1426 | 33.24 | 186 | 31.00 | 0.404 |
| Brazil | 1442 | 33.61 | 200 | 33.33 |  |
| Mexico | 1422 | 33.15 | 214 | 35.67 |  |
| **Age, years** |  |  |  |  |  |
| 18-30 | 2040 | 47.5 | 260 | 43.3 | 0.104 |
| 31-44 | 1646 | 38.3 | 256 | 42.7 |  |
| >45 | 613 | 14.3 | 84 | 14.0 |  |
| **Race** |  |  |  |  |  |
| White | 1886 | 44.72 | 271 | 45.70 | 0.529 |
| Black | 669 | 15.86 | 78 | 13.15 |  |
| Asian | 119 | 2.82 | 17 | 2.87 |  |
| Native Hawaiian or Other Pacific Islander | 3 | 0.07 | 0 | 0.0 |  |
| American Indian, Alaska Native | 81 | 1.92 | 9 | 1.52 |  |
| Other | 1459 | 34.60 | 218 | 36.76 |  |
| **Ethnicity** |  |  |  |  |  |
| Hispanic | 1926 | 47.15 | 282 | 47.88 | 0.740 |
| Non-Hispanic | 2159 | 52.85 | 307 | 52.12 |  |
| **Education, years** |  |  |  |  |  |
| ≤12 | 2105 | 49.27 | 287 | 48.07 | 0.339 |
| 13-15 | 1207 | 28.25 | 160 | 26.80 |  |
| ≥16 | 960 | 22.47 | 150 | 25.13 |  |
| **Marital Status** |  |  |  |  |  |
| Single, never married | 1934 | 45.24 | 260 | 43.55 | 0.886 |
| Married | 1454 | 34.01 | 212 | 35.51 |  |
| Cohabiting, living together | 511 | 11.95 | 67 | 11.22 |  |
| Divorced/separated | 362 | 8.47 | 56 | 9.38 |  |
| Widowed | 14 | 0.33 | 2 | 0.34 |  |
| **Smoking Status** |  |  |  |  |  |
| Current | 1038 | 24.20 | 147 | 24.50 | 0.984 |
| Former | 806 | 18.79 | 113 | 18.83 |  |
| Never | 2446 | 57.02 | 340 | 56.67 |  |
| **Circumcision** |  |  |  |  |  |
| No | 2687 | 63.75 | 389 | 64.83 | 0.605 |
| Yes | 1528 | 36.25 | 211 | 35.17 |  |
| **Sexual orientation** |  |  |  |  |  |
| MSW | 3565 | 88.11 | 526 | 88.85 | 0.800 |
| MSM | 121 | 8.90 | 51 | 8.61 |  |
| MSWM | 360 | 2.99 | 15 | 2.53 |  |
| **Alcohol Consumption, # drinks/month*** |  |  |  |  |  |
| Mean (SD) | 33.94 | (65.15) | 36.41 | (69.47) | 0.694 |
| Median (range) | 9.00 | (0-480) | 8.00 | (0-468) |  |
| 0 | 1024 | 24.17 | 141 | 23.90 | 0.790 |
| 1–30 | 1939 | 45.77 | 281 | 47.63 |  |
| 31-60 | 463 | 10.93 | 58 | 9.83 |  |
| ≥61 | 810 | 19.12 | 110 | 18.64 |  |
| **Number of Female Sex Lifetime Partners** |  |  |  |  |  |
| Mean (SD) | 17.06 | (45.46) | 19.61 | (50.31) | 0.417 |
| Median (range) | 8.00 | (0-1000) | 8.00 | (0-700) |  |
| 0 | 83 | 2.05 | 60 | 10.71 | **<0.001** |
| 1-3 | 967 | 23.84 | 126 | 22.50 |  |
| 4-18 | 1779 | 43.86 | 247 | 44.11 |  |
| ≥19 | 1227 | 30.25 | 127 | 22.68 |  |
| **Number of Male Sex Lifetime Partners** |  |  |  |  |  |
| Mean (SD) | 3.54 | (44.31) | 4.00 | (42.46) | 0.6450 |
| Median (Range) | 0.00 | (0-2000) | 0.00 | (0-750) |  |
| 0 | 3309 | 85.59 | 508 | 85.38 | 0.5630 |
| 1 | 155 | 4.01 | 29 | 4.87 |  |
| ≥2 | 402 | 10.40 | 58 | 9.75 |  |
| a. For categorical variables Chi-square and for continuous variables Wilcoxon rank-sum test were used to calculate p-values. | | | | | |
